# Knockdown of SETD5 inhibited glycolysis and tumor growth in gastric cancer cells by down-regulating Akt signaling pathway

**DOI:** 10.1515/biol-2022-0697

**Published:** 2023-10-24

**Authors:** Jing Shi, Litao Yu, Changhong Zhu, Haiyan Zhong

**Affiliations:** Department of Gastroenterology, Changzhou Tumor Hospital, No. 68 Honghe Road, Xinbei District, Changzhou, Jiangsu, 213031, China; Department of Obstetrics and Gynaecology, Changzhou Maternity and Child Health Care Hospital, Changzhou, Jiangsu, 213031, China

**Keywords:** gastric cancer, SET domain containing 5, proliferation, glucose consumption, glycolysis

## Abstract

Gastric cancer (GC) is the 5th most common cancer and the 3rd leading cause of cancer-related death worldwide. It is of great significance to study the underlying molecular mechanism of GC, and targeting glycolysis is a good strategy to treat GC. SET domain containing 5 (SETD5) contains a catalytic methyltransferase SET domain, which is known as a lysine methyltransferase that affects the progression of multiple cancers. However, its possible role in GC was still unclear. Here, we revealed that SETD5 was highly expressed in GC and was associated with a poor prognosis. Further through the *in vitro* experiments, we revealed that the downregulation of SETD5 inhibited the proliferation and migration of GC cells. Knockdown of SETD5 inhibited glucose consumption and glycolysis. Further studies have shown that SETD5 knockdown restrained the Akt signaling pathway. Therefore, we thought that SETD5 could act as a GC target.

## 1 Introduction

Gastric cancer (GC) is the 5th most common cancer and the 3rd leading cause of cancer-related death in the world [1]. Although great progress has been made in diagnostic methods and surgical procedures over the past few years, the high recurrence rate and the low 5-year survival rate of GC are still unsatisfactory [2,3]. Therefore, it is key to investigate the underlying molecular mechanism of the malignant progression of GC to improve the survival and reduce the recurrence rate of GC patients.

Metabolic reprogramming of tumor cells is usually caused by the Warburg effect [4]. Recent studies have found that rapidly proliferated cancer cells rely on glycolysis for energy needs, even when oxygen is available [5]. Although the efficiency of ATP produced by glycolysis is quite low, it can rapidly provide an energy supply for tumor cells and macromolecular substances synthesized by molecules, forming an acidic environment and promoting metastasis of tumor cells [6]. Therefore, targeting glycolysis is a good strategy for treating GC.

SET domain containing 5 (SETD5), which contains a catalytic methyltransferase SET domain, is known as a lysine methyltransferase [7]. SETD5 gene has been found to be mutated in patients with intellectual disabilities and autism spectrum disorders [8]. Loss of SETD5 in embryonic stem cells leads to impaired proliferation and differentiation, further altering gene expression [9]. The function of SETD5 in cancer is largely unexplored. SETD5 plays a critical role in multiple types of cancers [10,11,12,13]. SETD5 enhances the cytodryness of non-small-cell lung cancer (NSCLC) through the PI3K/Akt/mTOR pathway [13,14]. In addition, SETD5 mediates the glycolysis in breast cancer stem cell-like cells and promotes tumor growth [12]. However, its possible effects on GC are still unclear. TCGA data analysis found that SETD5 was highly expressed in GC and was associated with poor prognosis, but the function of SETD5 in GC was not clear. In this study, it was found that knockdown SETD5 inhibited the proliferation and motility of GC cells, as well as suppressed the glycolysis of GC cells. Further studies showed that the downregulation of SETD5 restrained the Akt pathway. Therefore, we thought that it could serve as a promising GC target.

## 2 Materials and methods

### 2.1 Bioinformatics

To be more objective about the genes under study, transcriptome data were obtained from The Cancer Genome Atlas databases. In addition, the expression level of SETD5 and its relation with the overall survival of GC patients were analyzed using GEPIA online platforms based on the TCGA database. Kaplan–Meier in the low- and high-risk groups was analyzed. The online platform GEPIA was used to analyze the expression of SETD5 and its relation with the overall survival of GC patients based on the TCGA database.

### 2.2 Cell culture and transfection

Human gastric cell lines GES-1 and GC cells (AGS, HS746T, MKN-45, and HGC-27) were all purchased from the Cell Bank of the Chinese Academy of Sciences. Gastric and GC cells were cultured with the complete medium (DMEM containing 10% FBS). Cells were seeded in 6-well plates with 1 × 10^5^ cells in each well. After 12 h of culture, Lipofectamine^®^3000 reagent (Invitrogen, Carlsbad, CA, USA) was used to co-transfect the cells with sh-NC, sh-SETD5, pcDNA-SETD5, and pcDNA, respectively. 5 μL Lipofectamine^®^3000 reagent and 5 μg shRNA plasmids were used in each well in 100 μL serum-free medium. 100 μL of the mixture was slowly added to the six-well plate, and the cells were incubated for 6 h. Then, western blotting was used to detect protein expression in the cells, and the transfection effect was verified for subsequent experiments.

### 2.3 Quantitative PCR

TRIzol (Invitrogen, Waltham, MA, USA) reagent was used. Then RNA was reverse-transcribed into cDNA using Moloney Murine Leukemia Virus Reverse Transcriptase (Promega, Madison, WI, USA). Fast Start Universal SYBR Green Master kit (Roche, Basel, Switzerland) was used for quantitative mRNA detection on the ABI StepOne system (Applied BioSystems, Foster City, CA, USA). The levels of targeted genes were determined by using the 2^–ΔΔCT^ method.

### 2.4 Western blotting

Total protein was extracted from cells using RIPA buffer, and then, the protein was quantitated by BCA reagent, separated by SDS-PAGE, and further transferred to the PVDF membrane. The proteins were blocked with TBST containing 5% milk for 1 h, and then, the corresponding primary antibodies were added and incubated at 4°C overnight. Primary antibodies included SETD5 (Abcam, ab204363; 1:1,000), SLC2A1 (Abcam, ab261869; 1:500), PFKFB3 (Abcam, ab181861; 1:1,000), HK2 (Abcam, ab273721; 1:1,000), LDHA (Abcam, ab52488; 1:1,000), and β-actin (Abcam, ab8226; 1:3,000), and then, secondary antibodies were incubated with membranes for 1 h and photographed after chemiluminescence. The reagents used in this were purchased from Wuhan Google Co., Ltd.

### 2.5 MTT assay

GC cells were plated into 96-well plates with 1,000 cell density and then maintained for 48 h. Cells were subsequently incubated with MTT for 4 h and then dissolved with 150 μL of DMSO. Then, the OD value was measured at 490 nm wave length by a microplate reader (BD).

### 2.6 Colony formation assay

Cells were plated into the six-well plates (500 cells per well) and maintained in media (10% FBS) for 10 days at 37°C. Then, cells were fixed with PFA for 15 min and then stained with 0.1% crystal violet for 20 min. Then, cells were photographed by an Olympus microscope.

### 2.7 Transwell assays

Cells were plated into the upper of Transwell chambers in a culture medium without serum. Subsequently, a medium containing 10% FBS was added to the bottom to stimulate motility. After 24 h, cells in the upper were removed, and the remaining cells were fixed and stained with crystal violet and photographed by an Olympus microscope.

### 2.8 Glucose intake test

The glycolysis levels of cells were detected according to the glucose intake kit (Abcam, ab136955) according to the relevant instructions.

### 2.9 Oxygen consumption rate (OCR) and extracellular acidification rate (ECAR) test

OCR and ECAR were detected on an XF24 Extracellular Flux Analyzer (Seahorse Bioscience, Billerica, MA) following the manufacturer’s guidelines. OCR and ECAR measurement was performed after the exchange of medium for 90 min. The inhibitors of the electron transport chain and oxidative phosphorylation were injected: oligomycin A (1 μM), CCCP (1.5 μM), rotenone (0.5 μM), and antimycin A (0.5 μM).

### 2.10 Statistics

GraphPad 5.0 software was used for the statistical analysis. Data were represented as mean ± SEM. Student’s *t*-test was used for the comparisons, and *p* < 0.05 was considered statistically significant.

## 3 Results

### 3.1 SETD5 was highly expressed in human GC tissues and cells and correlated with the prognosis

**Figure 1:**
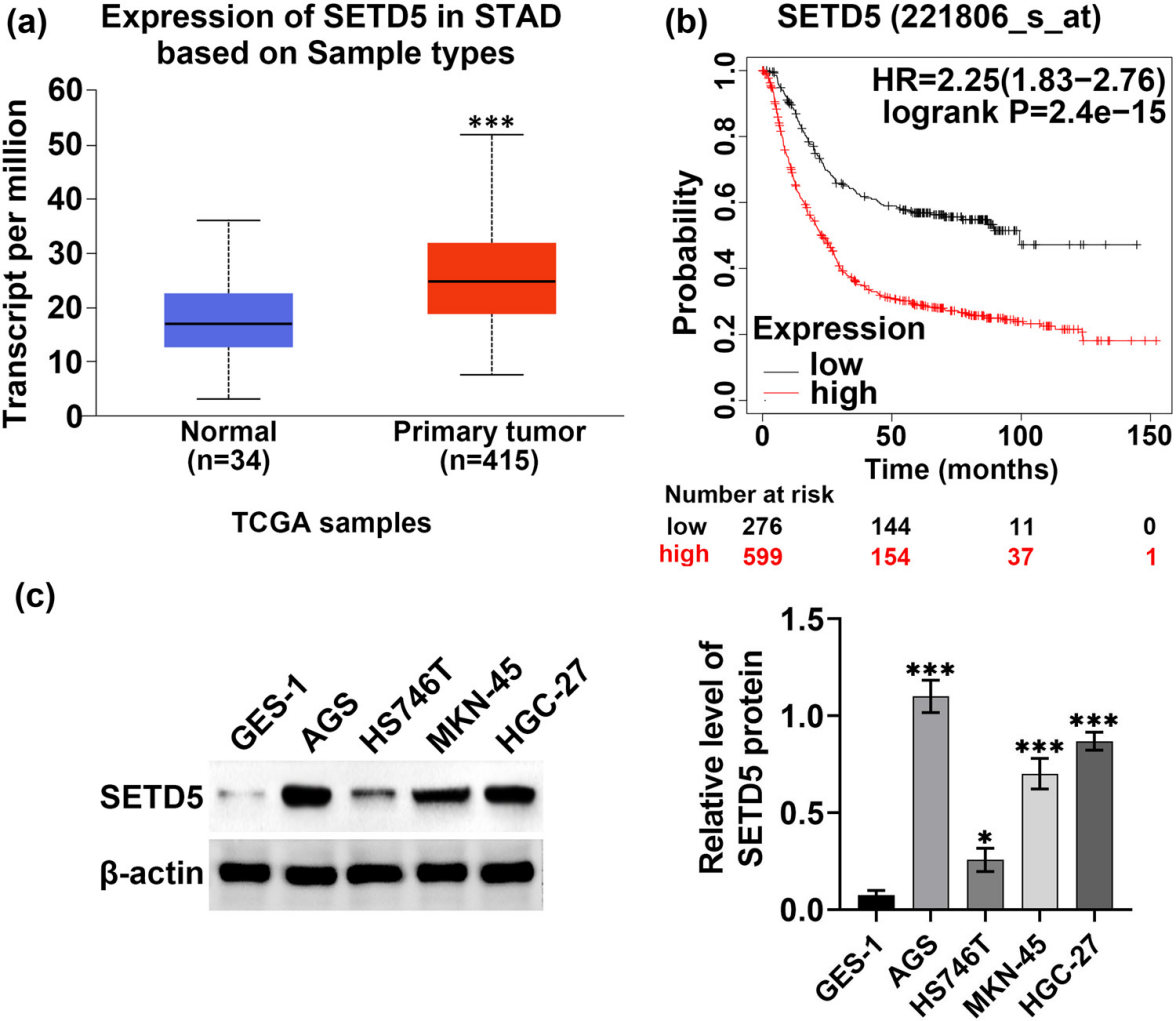
SETD5 was highly expressed in human gastric cancer tissues and cells and correlated with the prognosis of gastric cancer. (a) TCGA database showed the expression of SETD5 in gastric cancer tissues and normal tissues. (b) TCGA database showed the correlation between SETD5 expression and the prognosis of patients with gastric cancer. (c) Immunoblot showed the protein levels of SETD5 in cell lines including gastric cells GES-1 and GC cell lines (AGS, HS746T, MKN-45, and HGC-27). *p < 0.05, ***p < 0.001.

We first detected the expression of SETD5 in GC tissues. We analyzed the expression of SETD5 in GC tissues (*n* = 415) and normal tissues (34) according to the TCGA database. We found a high expression of SETD5 in the GC tissues of patients (Figure 1a). Further, through the clinical sample analysis, it was found that SETD5 expression was correlated with the prognosis of patients with GC (Figure 1b). Subsequently, we detected SETD5 protein levels in GC cells including AGS, HS746T, MKN-45, and HGC-27 cells, and normal gastric GES-1 cells. Through Immunoblot, we found high protein levels of SETD5 in GC cells compared to normal gastric cells (Figure 1c). The immunostaining assays also showed high protein levels of SETD5 in GC cells compared to normal gastric cells (Figure S1). Therefore, SETD5 was highly expressed in human GC tissues and cells and correlated with the prognosis.

### 3.2 SETD5 depletion suppressed viability as well as the motility of GC cells

**Figure 2:**
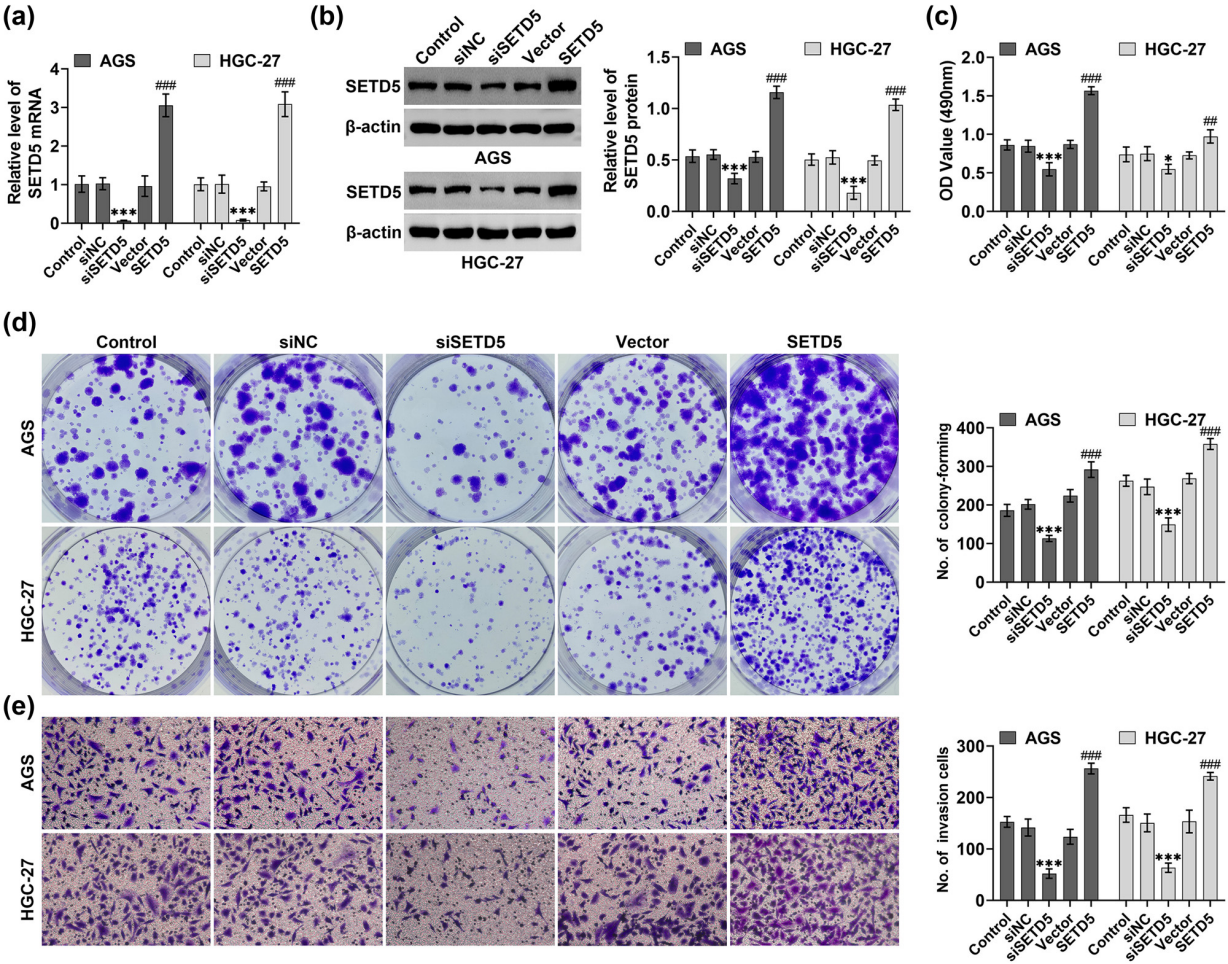
SETD5 depletion suppressed viability as well as the motility of GC cells. (a) qPCR showed the mRNA levels of SETD5 in AGS and HGC-27 cells upon the indicated transfection. (b) Immunoblot showed the expression of SETD5 in AGS and HGC-27 cells upon the indicated transfection. (c) MTT assays showed the OD value at 490 nm wavelength of AGS and HGC-27 cells upon the indicated transfection at 48 h. (d) Colony formation assay showed the viability of AGS and HGC-27 cells upon the transfection of indicated plasmids or siRNAs. (e) Transwell assay showed the effects of SETD5 on the invasion of AGS and HGC-27 cells upon the indicated transfection. ^##^ *p* < 0.01, ^###^ *p* < 0.001, SETD5 vs control; ***p < 0.001. siSETD5 vs siControl.

Subsequently, we detected the effects of SETD5 on the viability and motility of GC cells. The plasmids and siRNAs of SETD5 were transfected into AGS and HGC-27 cells to alter the expression of SETD5 in GC cells. Through qPCR and Immunoblot, we revealed that the transfection of SETD5 plasmids obviously increased its expression in AGS and HGC-27 cells, whereas the transfection of SETD5 siRNAs significantly suppressed the expression of SETD5 in GC cells (Figure 2a and b).

Through MTT assays, we found that SETD5 overexpression promoted the viability of AGS and HGC-27 cells, and its knockdown suppressed the viability of cells, with decreased OD490 value (Figure 2c). Through colony formation assays, we found that SETD5 overexpression increased the colony formation number of AGS and HGC-27 cells, whereas its knockdown decreased the colony numbers of GC cells (Figure 2d). Subsequently, we performed transwell assays to detect the effects of GC on cell motility. We noticed that SETD5 overexpression increased invasive cell numbers, whereas its downregulation decreased invasive cell numbers in AGS and HGC-27 cells (Figure 2e). Therefore, SETD5 knockdown suppressed viability and the motility of GC cells.

### 3.3 SETD5 knockdown inhibited aerobic glycolysis of GC cells

**Figure 3:**
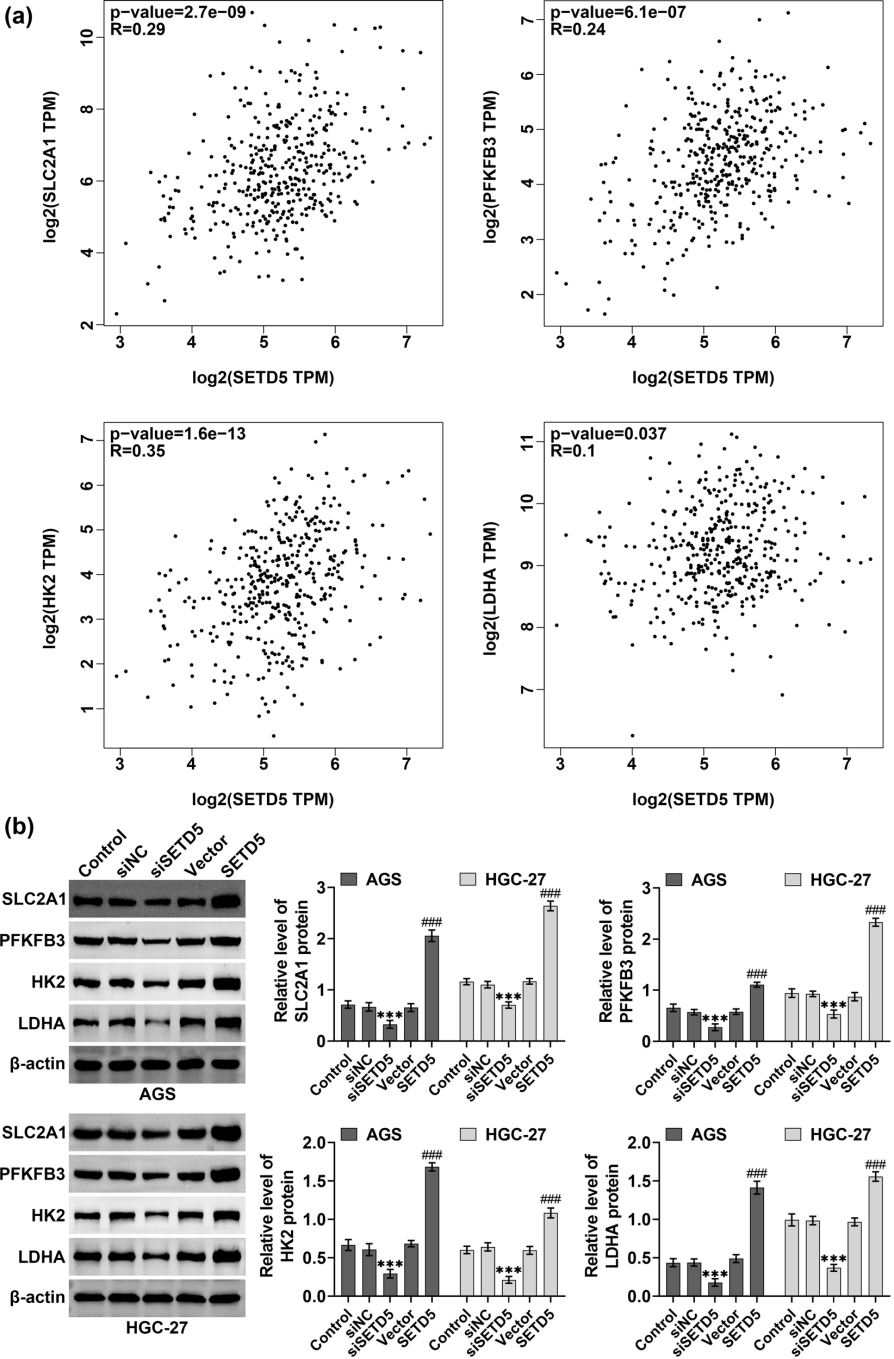
The expression of SETD5 is correlated with glycolysis of GC cells. (a) GEPIA database showed the correlation between SETD5 expression and the expression of glycolysis markers including SLC2A1, GLUT1, HK2, as well as LDHA in gastric cancer. (b). Immunoblot showed the protein levels of SLC2A1, GLUT1, HK2, as well as LDHA in AGS and HGC-27 cells upon the indicated transfection. ^###^ *p* < 0.001, SETD5 vs control; ***p < 0.001. siSETD5 vs siControl.

Then, we detected the effects of SETD5 on the aerobic glycolysis of GC cells. Through GEPIA analysis, we found that the expression of SETD5 was correlated with four aerobic glycolysis markers, including GLUT1, HK2, and LDHA (Figure 3a). We detected the expression of markers of aerobic glycolysis, including GLUT1, HK2, and LDHA in AGS and HGC-27 cells by western blotting upon the overexpression and knockdown of SETD5. We found that SETD5 overexpression increased the expression of GLUT1, HK2, and LDHA, and downregulation of SETD5 suppressed the expression of these proteins in AGS and HGC-27 cells (Figure 3b).

**Figure 4:**
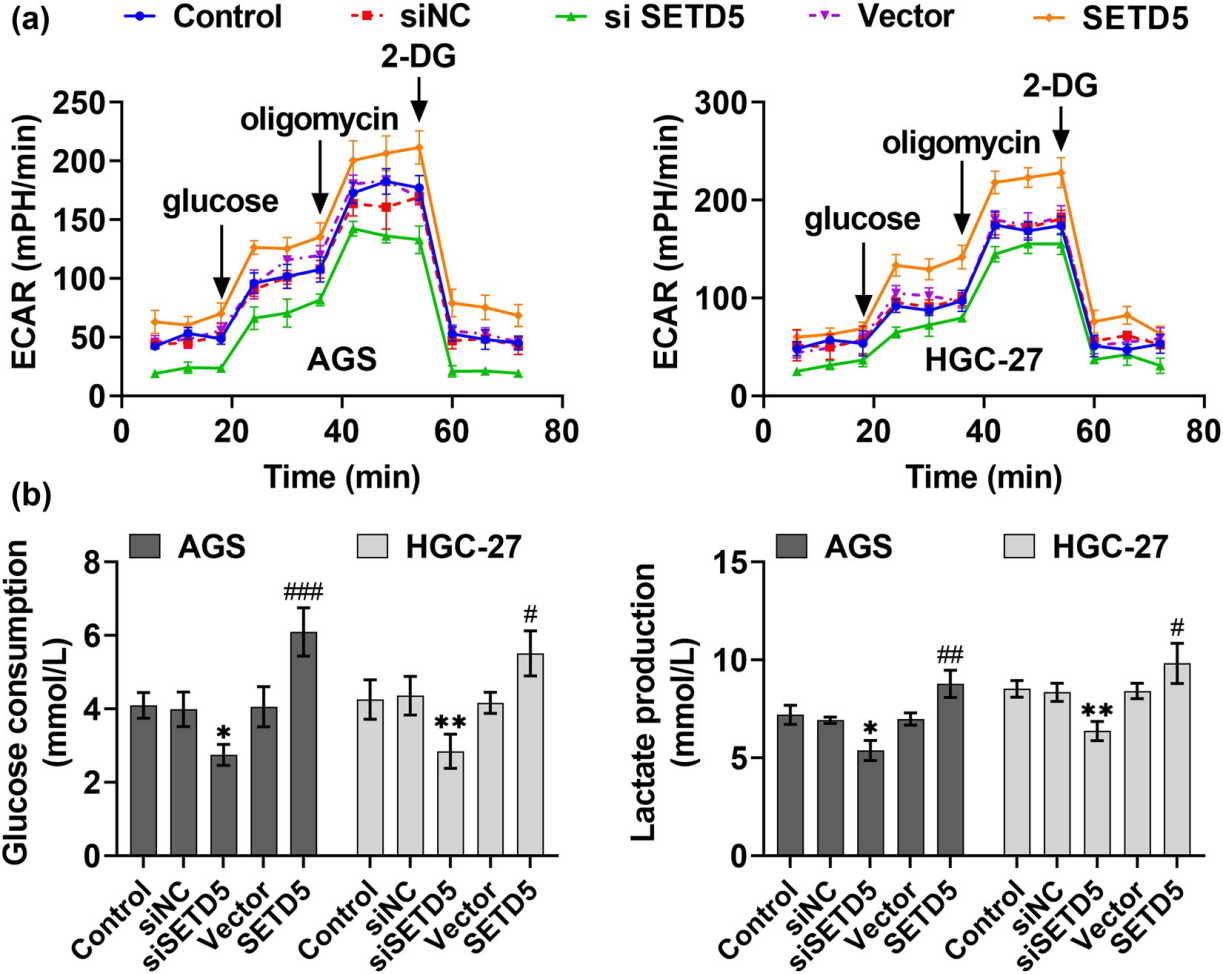
SETD5 ablation suppressed the glucose consumption in GC cells. (a) OCR and ECAR levels of AGS and HGC-27 cells upon the indicated transfection. (b) Glucose uptake assays showed the relative glucose uptake capacity of AGS and HGC-27 cells upon the indicated transfection. ^#^ *p* < 0.05, ^##^ *p* < 0.01, ^###^ *p* < 0.001, SETD5 vs control; *p < 0.05, **p < 0.01, ***p < 0.001. siSETD5 vs siControl.

We then performed OCR and ECAR assays and found that SETD5 overexpression enhanced OCR and suppressed ECAR in AGS and HGC-27 cells, whereas its knockdown suppressed the OCR and increased ECAR (Figure 4a). We subsequently performed glucose uptake assays, and the data confirmed that SETD5 overexpression promoted the glucose uptake in AGS and HGC-27 cells, whereas its knockdown suppressed the glucose uptake (Figure 4b). Therefore, SETD5 knockdown inhibited aerobic glycolysis of GC cells.

## 4 SETD5 knockdown suppressed the Akt pathway in GC cells

**Figure 5:**
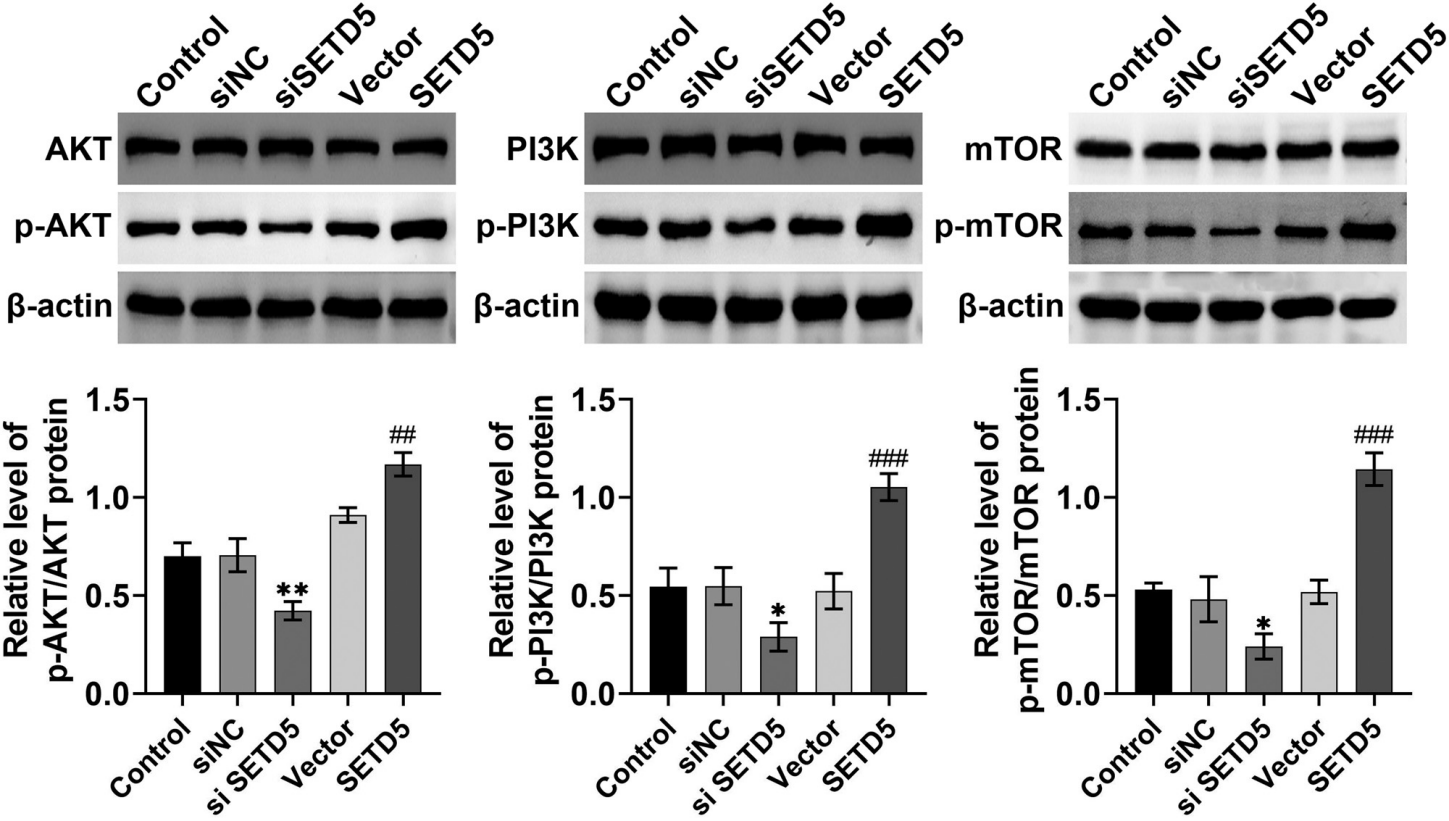
SETD5 knockdown suppressed the Akt pathway in GC cells. Immunoblot showed the protein levels of Akt, PI3K, and mTOR and the phosphorylation levels of Akt, PI3K, and mTOR in AGS and HGC-27 cells upon the indicated transfection. ^##^ *p* < 0.01, ^###^ *p* < 0.001, SETD5 vs control; *p < 0.05, **p < 0.01. siSETD5 vs siControl.

Subsequently, we investigated the possible mechanism underlying SETD5 promoting the progression of GC. Through Immunoblot, we noticed that the knockdown of SETD5 suppressed the phosphorylation of Akt, PI3K, and mTOR in AGS and HGC-27 cells (Figure 5). However, the overexpression of SETD5 significantly promoted the phosphorylation levels of Akt, PI3K, and mTOR in AGS and HGC-27 cells but did not affect the total protein expression (Figure 5). These data suggested that SETD5 activated the Akt pathway in GC cells.

### 4.1 SETD5 knockdown suppressed GC cell glycolysis and growth

**Figure 6:**
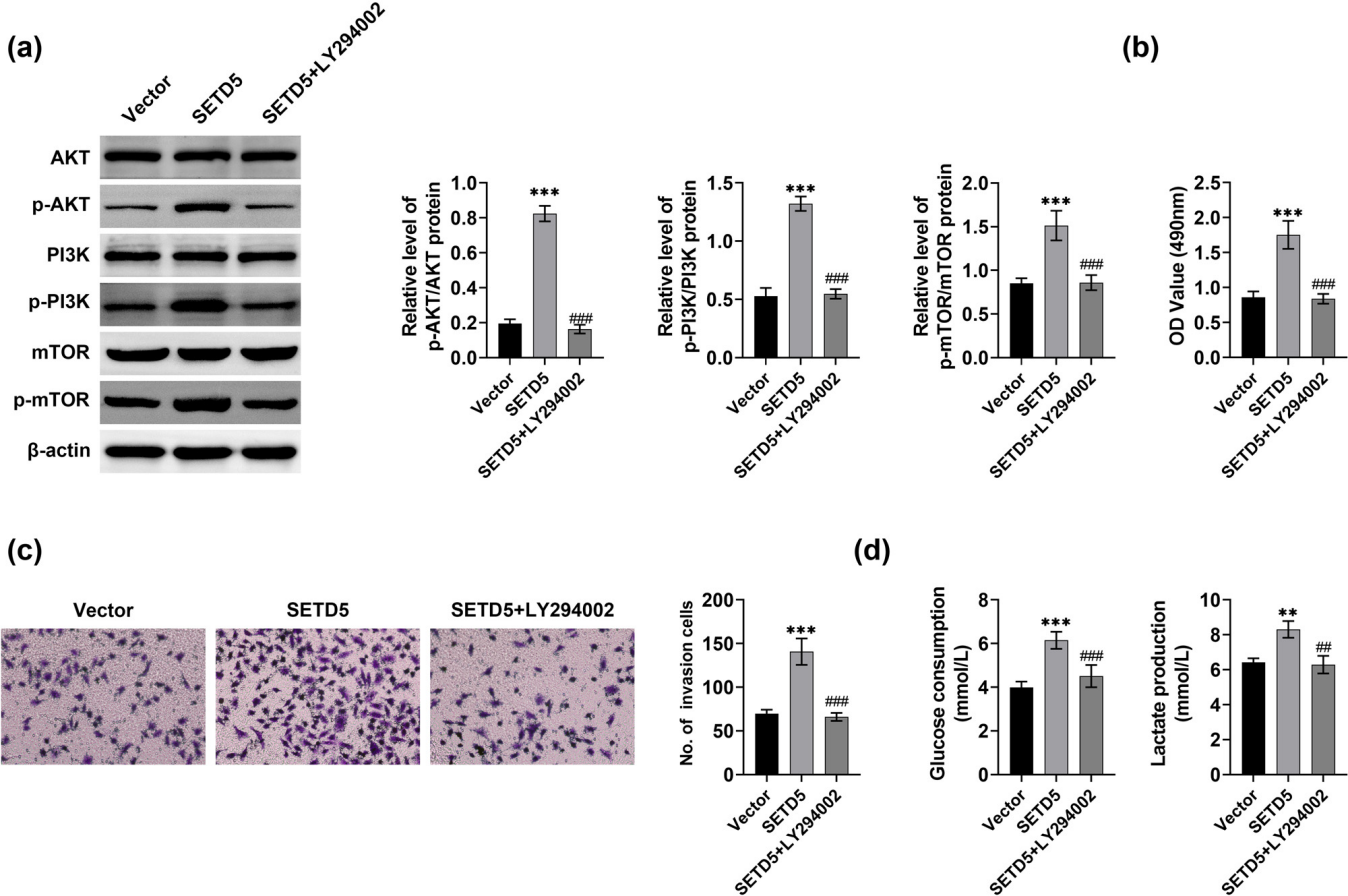
SETD5 knockdown suppressed GC cell glycolysis and growth. (a) Immunoblot showed the expression of AKT, p-AKT, PI3K, p-PI3K, mTOR, and p-mTOR in AGS cells upon the indicated treatment. (b) MTT assays showed the OD value at 490 nm wavelength of AGS cells upon the indicated treatment at 48 h. (c) Transwell assay showed the invasion capacity of AGS cells upon the indicated treatment. (d) Glucose uptake assays showed the relative glucose uptake and lactate production capacity of AGS cells upon the indicated treatment. **p < 0.01, ***p < 0.001. SETD5 vs Vector. ^###^ *p* < 0.001, SETD5 + LY294002 vs SETD5.

To further confirm the possible mechanism, we used the inhibitor of the Akt pathway, LY294002, to detect its effects on the glycolysis and growth of AGS cells. Through Immunoblot assays, we found that SETD5 overexpression increased the phosphorylation levels of mTOR, Akt, and PI3K in AGS cells, and the treatment of LY294002 suppressed the phosphorylation levels of mTOR, Akt, and PI3K in AGS cells (Figure 6a). MTT and transwell assays confirmed that SETD5 overexpression increased the proliferation and invasion of AGS cells, whereas the treatment of LY294002 attenuated the promoted proliferation and invasion of AGS cells (Figure 6b and c). We further found that the treatment of LY294002 attenuated the promoted relative glucose uptake and lactate production capacity in AGS cells (Figure 6d). Therefore, SETD5 knockdown suppressed GC cell glycolysis and growth.

## 5 Discussion

GC is a common malignant tumor in the digestive system, with occult onset and no specific symptoms [15]. The incidence of GC ranks the first in China, and about 170,000 people die from GC every year, which is a serious threat to people’s health [15]. The survival rate of early GC is high, but once it reaches the advanced stage, even with comprehensive treatment, the survival rate is still less than 30% [16]. The occurrence of GC is caused by multiple types of factors, and to improve survival, it is still necessary to have a detailed and in-depth understanding of its pathogenesis [16]. In this study, TCGA data analysis showed that SETD5 was highly expressed in GC and was associated with a poor prognosis. We therefore thought that it could serve as a promising GC target.

SETD5 is an important methyltransferase that has been found to be abnormally expressed in multiple tumors, correlated with the patient prognosis, and involved in the regulation of tumor progression and metastasis [9,12,14,17]. Downregulation of SETD5 restrained the tumorigenicity of the hepatocellular carcinoma (HCC) cells [18]. SETD5 mediated the glycolysis in breast cancer stem-like cells and promoted tumor growth [12]. Similarly, here, we also found that SETD5 knockdown inhibited glucose consumption and glycolysis in GC cells. SETD5 contributed to tumor cell invasion and was associated with a poor prognosis in NSCLC patients [13]. Through MTT, colony formation, and transwell assays, we found that SETD5 knockdown inhibited GC cell proliferation and migration. Our data therefore showed the involvement of SETD5 in GC progression.

One of the most common metabolic changes in tumor cells relative to normal cells is aerobic glycolysis, also known as the Warburg effect [4]. This effect plays an important role in the development of GC. In contrast to well-differentiated normal cells, which rely on the oxidation of pyruvate to produce energy for physiological functions, rapidly proliferating tumor cells rely on glycolysis for energy needs, even when adequate oxygen is available [4]. Although the efficiency of ATP produced by glycolysis is low, it can rapidly provide an energy supply for tumor cells and macromolecular substances synthesized by molecules, forming an acidic environment and promoting tumor cell metastasis [4]. Therefore, identifying the key mediators of the glycolysis pathway will provide effective strategies for the diagnosis and treatment of GC. By analyzing ATP level, glucose consumption, lactic acid production, OCR, and ECAR, we found that SETD5 knockdown inhibited the glucose consumption of GC cells.

Through Immunoblot assays, we found that the downregulation of SETD5 inhibited the Akt pathway in GC cells. This pathway affected multiple cellular processes in tumor cells, such as proliferation, motility, drug resistant, autophagy, and glucose consumption [19,20]. Urolithin A suppressed tumor progression and induced autophagy in GC via the Akt pathway [21]. The Akt pathway was also involved in P2RY2 activation and stimulated the proliferation and metastasis of GC [22]. These studies confirmed that the Akt pathway could serve as a promising GC target.

In summary, we revealed that SETD5 was highly expressed in GC and was associated with a poor prognosis. Downregulation of SETD5 inhibited the proliferation and motility of GC cells, as well as the glycolysis. Further studies have found that SETD5 knockdown restrained the Akt signaling pathway. Therefore, we thought that SETD5 could act as a potential therapeutic target for the treatment of GC.

## Supplementary Material

Supplementary Figure
