# Supplementary material for: Knockdown of SETD5 inhibited glycolysis and tumor growth in gastric cancer cells by down-regulating Akt signaling pathway
Source: Open Life Sci. 2023 Oct 24;18(1):20220697. doi: 10.1515/biol-2022-0697 (PMC10628568; doi:10.1515/biol-2022-0697)
Supplement: Supplementary Figure [file biol-2022-0697-sm.pdf]

Supplementary material

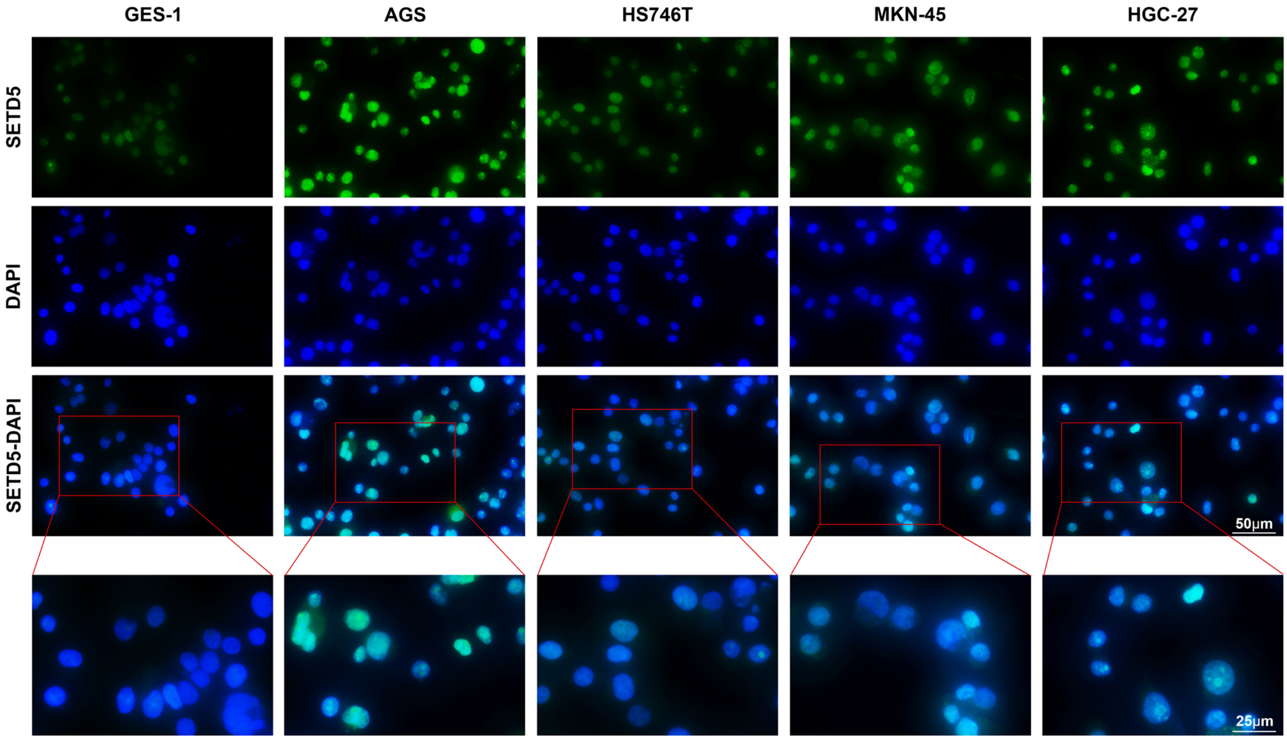

Figure S1: SETD5 was highly expressed in gastric cancer cells. Immunostaining assay showed SETD5 was highly expression in AGS, HS746T, MKN-45, and HGC-27 cells compared to the normal GES-1 gastric cells. The larger image was shown at the bottom.
